# Scanning electron microscopy examination of needle tips after different procedures of deep dry needling in humans

**DOI:** 10.1038/s41598-018-36417-w

**Published:** 2018-12-19

**Authors:** Emilio J. Poveda-Pagán, Sergio Hernández-Sánchez, Luis Rhys-Jones-López, Antonio Palazón-Bru, Carlos Lozano-Quijada

**Affiliations:** 1 Centro de Investigación Traslacional en Fisioterapia, Department of Pathology and Surgery, Miguel Hernández University, San Juan de Alicante, Alicante, Spain; 2 Department of Clinical Medicine, Miguel Hernández University, San Juan de Alicante, Alicante, Spain

## Abstract

The aim of this study was to evaluate the tips and the surface conditions of two types of needles with different quality and their possible alterations after performing different needling on human beings. A total of 160 needles from AguPunt brand were examined. Surface conditions (lumps and scratches) and tip of the needles after needling procedures in humans were tested using a JEOL JSM-6360LV microscopy device. Additionally, a group of physiotherapists assessed the use of both types of needles in clinical practice using a self-reported questionnaire. Both types of needles, after performing different needling on human beings, kept the needle tips well preserved although the dry needle (Type B) suffered very little deformation even touching the bone of the scapula 10 times versus acupuncture needle (Type A), which were deformed slightly. The surface conditions revealed irregularities and scratches in both types of needles but the tips of Type A suffered more damage after different procedures (Odds ratio = 0.04,95% CI:0.01–0.13, p < 0.001). The cellular tissue adhered to the surface was similar in both types of needles and the questionnaire about clinical practice of both types of needles showed that Type B seemed easier than Type A when the physical therapist penetrated the skin and when the needle went out the skin.

## Introduction

Acupuncture and dry needling are a therapeutic modalities that involves inserting needles into pathological parts of muscles^1^ and they are effective in the short term for pain relief, increasing range of motion and improving quality of life when compared to no intervention/sham/placebo^2^. Acupuncture^3–5^ and dry needling^1,6^ are the most commonly used techniques for the treatment of myofascial pain caused by the myofascial trigger points, which are identifiable as a hyperirritable focus located in a tense band of muscular fibers, and are often responsible of hyperalgesia and allodynia. Furthermore, active trigger points can produce sensorial, autonomic and motor dysfunctions (weakness, stiffness, restricted range of motion and alteration in the normal patterns of motor activation)^7^.

Steinbrocker^8^ is considered the first author to have described the benefits of needling to control myofascial pain, followed by numerous studies and authors, such as Travel and Simons, who described in detail the technique of the application of dry needling^6,7^.

Dry needling consists of the use of the mechanical stimulus of the needle, without introducing or extracting any substance, with the aim of eliminating or inactivating the myofascial trigger point^1,6^. Different types of dry needling techniques have been described. Some of these consist of repeated rapid introductions of the needle in the area of the myofascial trigger point^6,7^ and Hong’s technique^9–11^, thus obtaining therapeutic benefits^7^.

Acupuncture and dry needling is a technique used by physical therapists worldwide, is recognized by prestigious organizations such as the Cochrane collaboration and recommended as a treatment for people with chronic lumbar pain^1^.

Initially, the needles used to carry out the dry needling technique on the trigger points were hollow needles, but later solid needles were used. These needles originated in 1977 in the West Midlands, UK^12^. Acupuncturists performed their interventions with nondisposable needles, these being sterilised after each usage, but it was after an outbreak of hepatitis B that the UK Ministry recommended the use of single-use disposable needles to avoid any source of contagion. In 1978 the first disposable acupuncture needle was manufactured. But the first batches were very costly and it was then that China decided to take up the challenge of reducing production costs by combining automation with low labour costs^13^.

Nowadays, the use of these disposable needles has expanded all over the world, with many companies producing them at a reasonable price. But the reduction in production costs could put at risk the quality of the needle. In 2002, Hayhoe *et al*.^13^ studied three brands of needles, some manufactured in China, some in Japan and others in the USA, by scanning electron microscopy (SEM). After analyzing the SEM images they discovered that no needle was perfect and most of them showed significant faults.

Another study carried out in 2013 by Xie *et al*.^14^ using SEM, analyzed two of the most widely used brands on the market, one from China and another from Japan, by randomly choosing 10 needles of each brand and performing interventions in gel pads from a UNICO Needling Practice Kit as a simulation of human tissue. They observed that one of the brands showed a clear difference in the finish of the needles, which showed surface irregularities or distorted points. Moreover, some needles had foreign bodies, which disappeared after the insertion, possibly remaining in the tissue.

To the best of our knowledge there is no study analyzing the tips of the two types of needles (Type A and B) with different quality from one of the most widely brands used (AguPunt) and how they can be deformed after performing some common interventions in the treatment of trigger points. Hence, the main aim of this study was to evaluate, using SEM, the tips and the surface conditions of the deep dry Type B and Type A from AguPunt brand and their possible alterations after performing different needling on human beings. Secondly, the study aimed to find the opinion of physical therapists regarding the performance of both Type A and Type B from AguPunt brand in clinical practice.

## Materials and Methods

This research was designed as a prospective observational study to analyze the tips of two different types of needles (Type A and Type B) after performing different needling on volunteer human beings. The study was carried out in the physical therapy laboratory of the Center of Translational Research in Physiotherapy at the University Miguel Hernández of Elche (Alicante).

### Types of needles

In this study we have investigated two types of needles after dry needling in humans: Type A (hereafter called Type A1 to Type A4) and Type B (hereafter called Type B1 to Type B4). Prepacked, sterile, single-use, disposable needles from one of the most widely brands used (AguPunt).

Designed by the manufacturer, Type A and Type B are needles that can be used for different needling techniques indistinctly (acupuncture and dry needling). Type A is used more for acupuncture and Type B has been created to perform the more specific deep dry needling treatment. Both types of needles are made of stainless steel, with the same nominal shaft diameter and lengths of 0.32 and 40 mm, respectively. However, according to the manufacturer, the characteristics of their compositions are different: Type A are composed of surgical steel and Type B are composed of steel class I, type II surgical stainless steel. Furthermore, there is a difference in the quality of two type of needles from the same manufacturer and the main difference between them is the triple polished and triple lubricant cover the Type B while Type A have two layers of oil. Despite the manufacturer’s instructions, recent investigations have studied the composition of Type A and Type B and found that molybdenum was not present in these types of needles^15^.

To examine the surface conditions, 160 needles were tested. Eighty-eight Type A and seventy-two Type B from AguPunt brand were indiscriminately selected from different batches of the commercial product. The use-by dates of the batches were May 2024 for Type A needles and March 2024 for Type B needles, approximately eight years after the time when all tests were completed (September 2017). Finally, the analyzed needles were randomly selected using the Microsoft Excel program for the final selection.

### Procedure of needle insertion and needling manipulation

Both types of needles were manipulated by a physiotherapist with more than 15 years of clinical experience in the management of trigger points using manual palpation and deep dry needling techniques. This aspect was a limitation and will produce straight bias technically. In addition, he had experience as a professor in postgraduate dry needling courses at the university. The volunteer human subjects’ interventions for Type A or Type B were randomized using the Microsoft Excel program. The study protocol was approved by the local Ethics Committee of the University Miguel Hernandez of Elche (DPC.EPP.01.17). Informed consent was developed in accordance with the requirements of the Declaration of Helsinki and was signed for all subjects before needle insertion.

The reference position for dry needling puncture (Type A and Type B) was a standardized prone position, with the head resting in the hole of the table. The needled area was swabbed with 90° alcohol and intramuscular needling was then carried out via deep dry needling (according to the Western medicine principles) into the selection area^10,16–20^. Six different procedures were performed with needles in 30 different subjects (18 women and 12 men; aged 18 to 33 years old with mean age ±SD, 22.7 ± 5.9 years): 1 skin entry (2–3 mm) in the upper trapezius area; 5 skin entries (2–3 mm) in the upper trapezius area; 10 deep dry needling entries around the MTrP1 area of the soleus muscle (40 mm); 10 deep dry needling entries around the MTrP area in L1 of the longissimus lumbar (30–40 mm); 1–2 inputs and outputs touching the scapulae; and 10 inputs and outputs touching the scapula(30–40 mm). Immediately after the needling intervention care was taken to avoid contamination. The needles were inserted into a cork structure to avoid damaging the tip and were analyzed with SEM.

### SEM characterization

**Figure 1:**
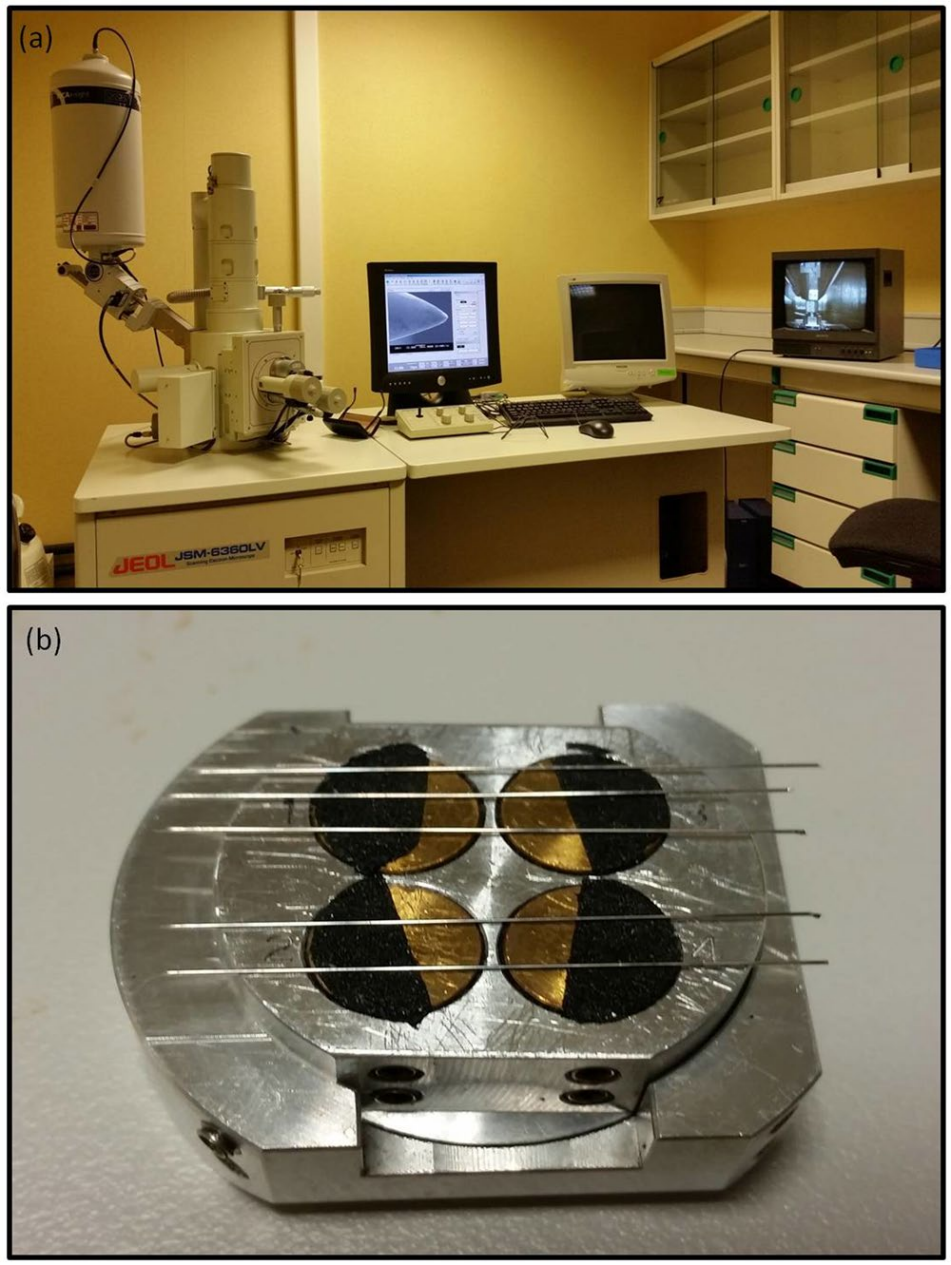
Images of scanning electronic microscopy and aluminum plate. (**a**) Scanning electronic microscopy. (**b**) Aluminum plate.

To examine the surface conditions of the Type A and Type B after needling, a JEOL JSM 6360LV SEM (Fig. 1a) was used with a high resolution of 3.0 nm at 30 kV, although in our study we used a resolution of 20 Kv. In the initial trials with the test needle the voltage was varied to find the optimum value, and magnifications of x200, x400, x1000, x2000 and x5000 were studied for the longitudinal cut. The highest magnification (x5000) gave a too close-up view of the point, with insufficient length of the rest of the needle tip. However, when we analyzed the transversal cut of needles, the magnifications used were of 200x, 500x, 1000x, 5000x and 10000x.

In order to check the firmness of the lumps or other alien materials attached to the needle surfaces, several other randomly selected needles from each brand were cleaned in an ultrasonic washing machine (Euronda Eurosonic 3D) for 10 minutes. We did not find differences in the needle surface between needles that were cleaned with the ultrasonic machine and needles that were not cleaned.

The needles were used to conduct the needling tests on patients. After the needling tests, SEM images of the randomly selected needles were taken to check the surface conditions of the needle tips and shafts, with a view to identifying defects such as scuffs, scratches, lumps and irregularities. Needle examinations were carried out by a blinded SEM microscope technician. Other researchers prepared the needles in an aluminum plate, indicating the name of the images taken. Care was taken to avoid contamination during preparation: needle tips were mounted directly with no coating, so that any loose dirt or contamination from the mounting process would have been blasted off the specimen. A distance of 10 mm was used to take photomicrographs of needle tips at both low (x400) and high (x2000) magnification. For the SEM examination, the as-received needles were cut at the handle and then placed on an aluminum plate (Fig. 1b), with their handles fixed by conducting resin. The block was placed in the electron microscope chamber and was then sealed in the vacuum chamber of the SEM device. Then, the needles were examined using the SEM. For acupuncture needles, these specimens were designated as Type A needles (Type A1, Type A2, Type A3 and Type A4) and Type B needles (Type B1, Type B2, Type B3 and Type B4).

### Qualitative evaluation of both types of needles by professionals

An ad hoc questionnaire was created to analyze the perception of needling manipulation in clinical practice of both types of needles (Type B and Type A) by physical therapists. The questionnaire was self-completed by the physical therapists. The questionnaire asked about the number of hours’ training in dry needling and acupuncture and about the most common technique use with Type B, indicating the name of techniques, such as Hong^11,17,21^, Gunn^22^ or Baldry^23,24^. Then, five items (repeated for both types of needles), were about the ease of use of different techniques and procedures: Hong’s technique, Gunn’s technique, Baldry’s technique and about ease of penetrating the needle into the skin and ease of withdrawing from the skin. The items were scored with five options: Strongly agree, agree, neither agree nor disagree; disagree, strongly disagree.

### Statistical methods

We estimated a logistic mixed regression model in order to compare the analyzed outcomes in our needles, adjusted by the gender of the patient. Using these models, we obtained the adjusted odds ratio (OR) for the type of needles. All the calculations were performed with an error type I of 5% and the statistical software was R 3.3.3.

## Results and Discussion

**Figure 2:**
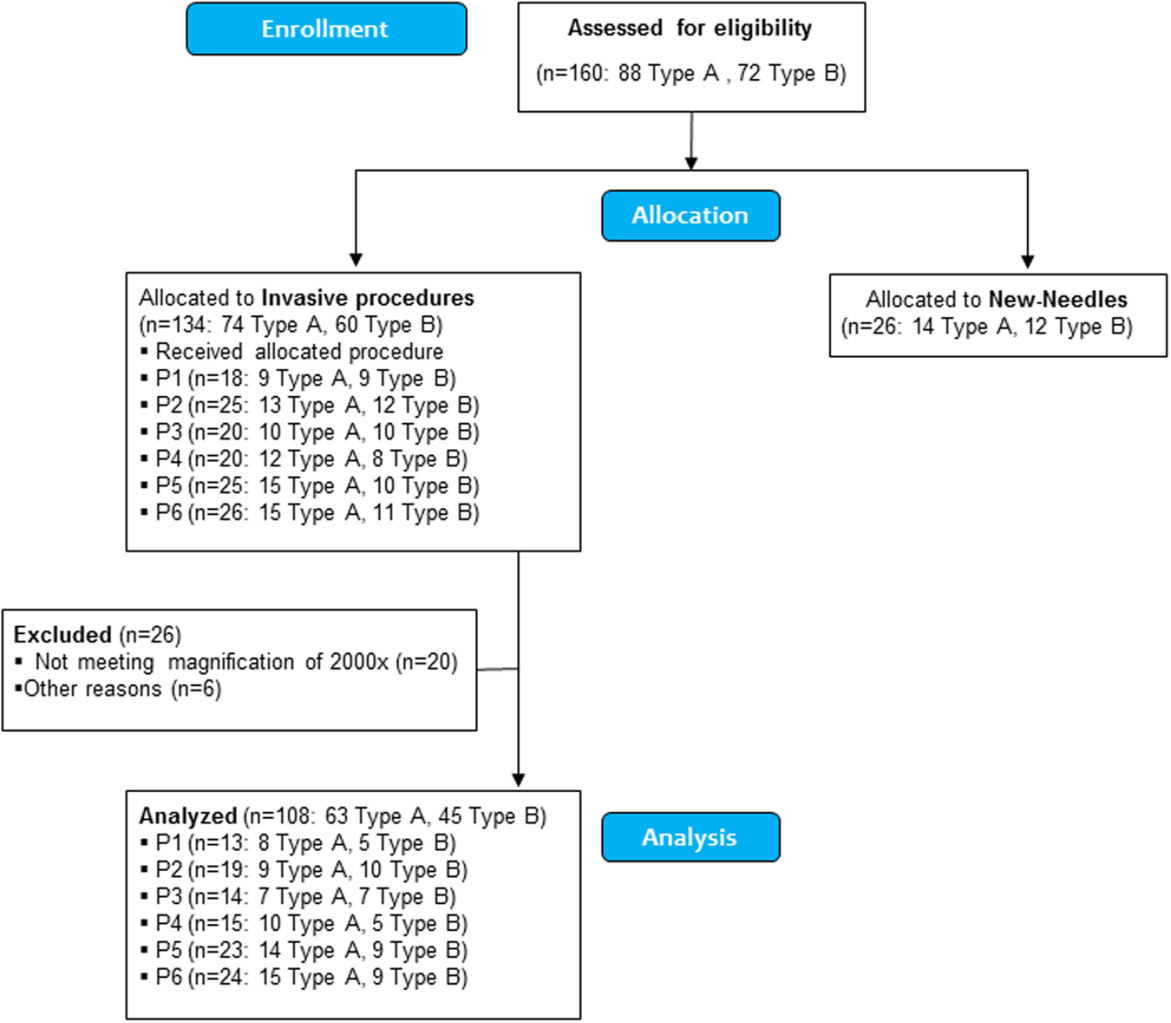
Flow diagram needles. Type A: Acupuncture needles; Type B: Dry needling needles; P1: 1 Skin entry; P2: 5 Skin entries; After 10 fast in and fast out Hong’s technique in soleus muscle; P3: After 10 fast in and fast out Hong’s technique in longissimus muscle; 1–2 Touching scapulae bone; 10 Touching scapulae bone.

One hundred and sixty needles from AguPunt brand (88 Type A and 72 Type B) were tested by SEM. First, twenty-six new needles were analyzed by SEM (14 Type A, 12 Type B) to know the basal state. Secondly, one hundred and thirty-four from invasive procedures were tested by SEM. Twenty-six needles were excluded and only one hundred-eight were statistically analyzed (63 Type A, 45 Type B) (Fig. 2).

There were significant statistical differences between tips of Type B and Type A (OR 0.04 = 95% CI: 0.01–0.13, p < 0.001), after performing different needling on human beings. Type B kept the needle tips well preserved in all procedures, even touching the bone of the scapulae 10 times versus acupuncture needles, which were deformed slightly. There were no significant differences with lumps (OR 0.95 = 95% CI: 0.38–2.64, p = 0.906) and scratches in both types of needles. The statistical analysis of the scratches could not performed because there was a coincidence in most of Type A and Type B.

### SEM observation of new needles: Type A and Type B

**Figure 3:**
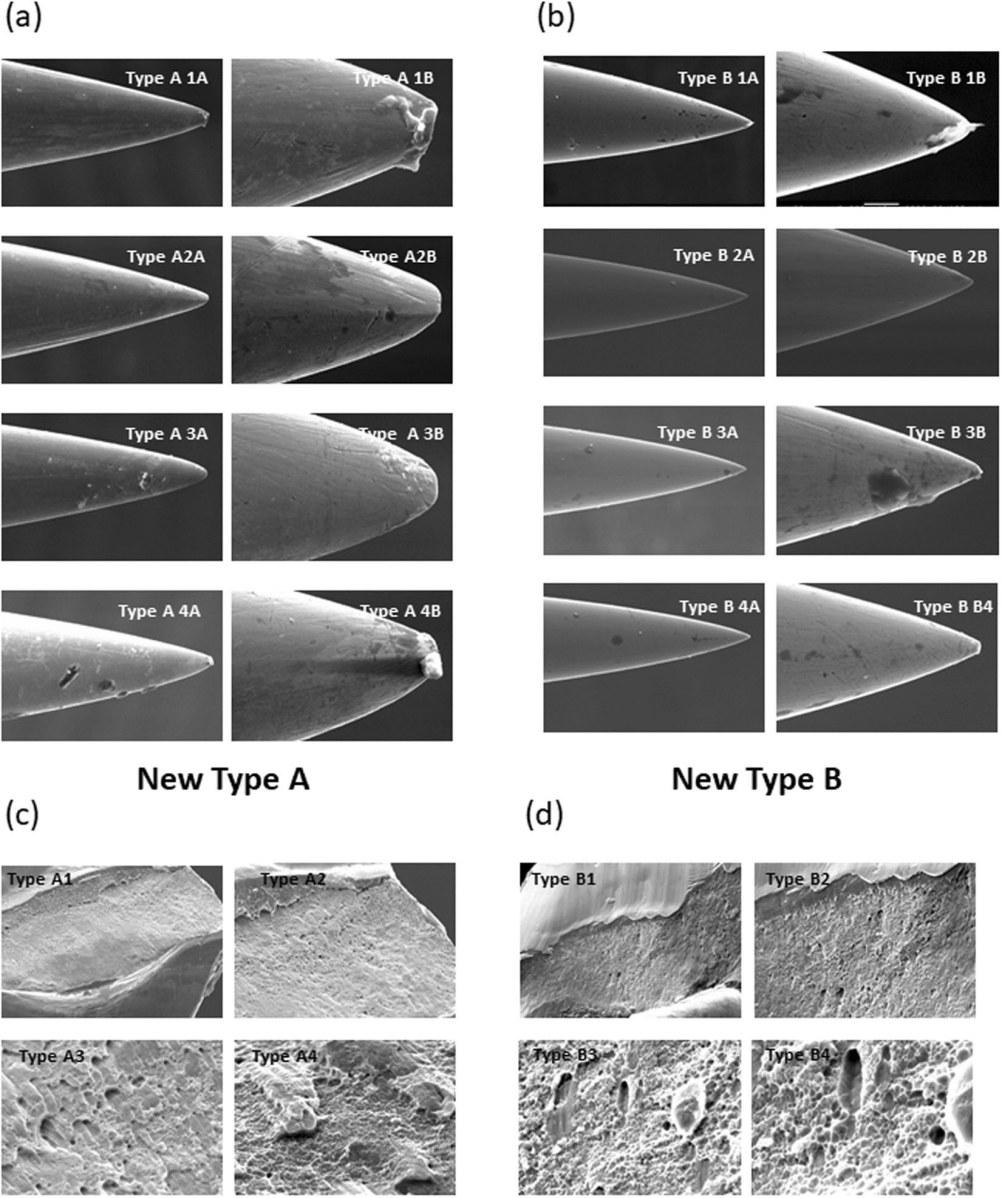
Scanning electronic microscopy images of new acupuncture and dry needles. (**a**) Type A1A, Type A2A, Type A3A, Type A4A: Acupuncture needles (magnification 400x); Type A1B, Type A2B, Type A3B, Type A4B: Acupuncture needles (magnification 2000x). (**b**) Type B1A, Type B2A, Type B3A, Type B4A: Dry needles (magnification 400x); Type B1B, Type B2B, Type B3B, Type B4B: Dry needles (magnification 2000x). (**c**) Type A1, Type A2, Type A3, Type A4: Acupuncture needle with transversal cut (magnification 500x, 1000x, 5000x, 10000x, respectively). (**d**) Type B1, Type B2, Type B3, Type B4: Acupuncture needle with transversal cut (magnification 500x, 1000x, 5000x, 10000x, respectively).

The SEM images of the randomly selected new Type A and Type B are shown in Fig. 3a,b. We observed that some Type A were not perfect and most of them showed minute faults, but not serious ones. The analysis of the tip and transversal cut showed a significant difference between the two types of needles. For Type A (Fig. 3a), with a magnification of 400x, there were no differences between them in the tip, but two exhibited impurities (Type A3A and Type A4A). A magnification of 2000x in the Type A tips exhibited scratches, blunt ends, lumps, attachments and irregularities (Type A1B, Type A2B, Type A3B and Type A4B). With regard to the Type B needles (Fig. 3b), they exhibited a relatively smooth and sharp end. Generally, the Type B characteristics were similar with a magnification of 400x and 2000x. Only in two Type B (Type B1B and Type B3B) could a few attachment be observed.

Hayhoe *et al*.^13^ analyzed three different brands of needles using electron microscopy and 52 electron micrographs were taken of 31 individual needles from 11 different types. They suggested that most manufacturers needed to reassess their quality control procedures. Fortunately, our results suggested that nowadays new Type A and Type B needles have better quality control.

The SEM images of the transversal cut are shown in Fig. 3c,d. No significant differences between the two types of needle were observed. Only the magnification of 10000x showed some differences. Type B (Fig. 3c) looked like a more porous material than the Type A (Fig. 3d) but this aspect is not important in clinical practice.

### Surface conditions of needles after acupuncture manipulation

#### After 1 or 5 skin entries

**Figure 4:**
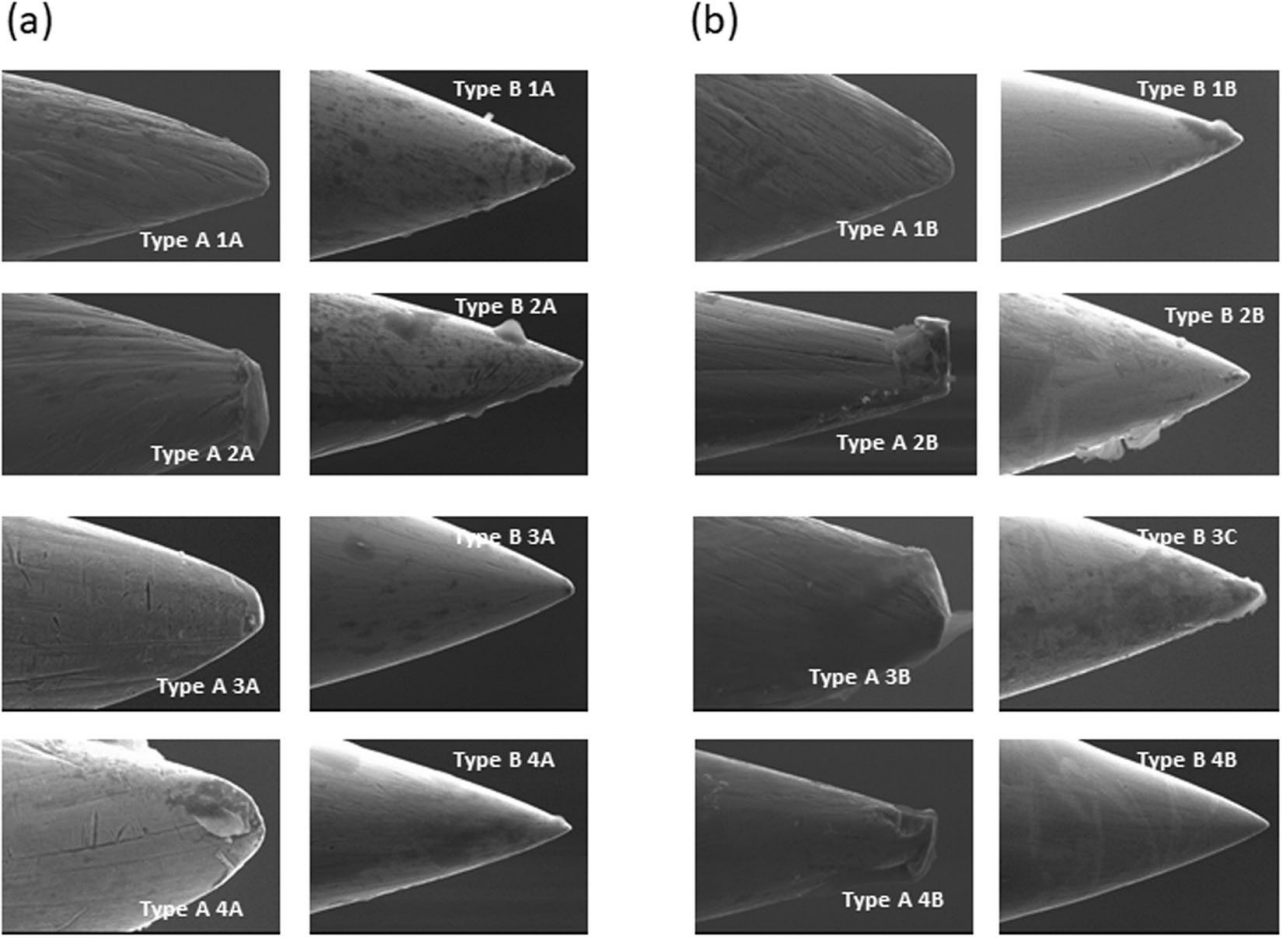
Scanning electronic microscopy images of acupuncture and dry needles after 1 and 5 skin entries. (**a**) Type A1A, Type A2A, Type A3A, Type A4A: Acupuncture needles after 1 skin entry (magnification 2000x); Type B1A, Type B2A, Type B3A, Type B4A: Dry needles after 1 skin entry (magnification 2000x). (**b**) Type A1B, Type A2B, Type A3B, Type A4B: Acupuncture needles after 5 skin entries (magnification 2000x); Type B1B, Type B2B, Type B3B, Type B4B: Dry needles after 5 skin entries (magnification 2000x).

The relevance of this study was that, previously, the surface of needle tips was not tested with SEM after dry needling in humans. The SEM images of the randomly selected Type A and Type B after one or five skin entries are shown in Fig. 4a,b. After 1 skin entry, Type A and Type B needles had impurities (Type A4A, Type B1A and Type B2A). After one skin entry, neither type of needle tips was deformed. However, the Type A surface exhibited scratches but Type B needles exhibited a smoother surface with fewer impurities than Type A. We observed cellular tissue adhered to the surface of both types of needles (Type A4A, Type B1A and Type B2A).

After five skin entries, the surface conditions of Type A showed scratches. However, Type B showed a smooth surface. An important aspect was that the two types of needles accumulated adhered cellular tissue with one skin entry (Type A2B, Type A3B, Type A4B, Type BI1B, Type B2B and Type B3B). Furthermore, the tip of the Type B did not deform after the five entries, although the Type A needles appeared to undergo a slight deformation with blunt ends (Type A2B, Type A3B and Type A4B). Unlike our work with humans, Xie *et al*.^14^ analyzed Type A surface conditions with SEM and other physical properties from two of the most popular brands widely used in many countries, after undergoing a standard manipulation with an acupuncture needling practice gel. The SEM images revealed significant surface irregularities and inconsistencies at the needle tips. Our results suggest that the end tip and surface conditions of the needles we used were better than those of the needles analyzed in Xie *et al*.’s study, especially the Type B needle tips’. Type A showed a worse surface and tip than Type B and this aspect would support the sentence that the needling sensation (de-qi) was more likely to occur if the tip was irregular. So it is possible that a perfectly smooth Type A might not be as effective clinically^13^.

Davis *et al*.^25^ tested the feasibility of quantifying acupuncture needle manipulation using motion and force measurements in humans, but did not analyze the needles with SEM. Rajabi *et al*.^26^ realized and evaluated microneedle patches with two different elasticities of the base substrate but did not analyze surface conditions using SEM.

#### After 10 fast in and fast out – Hong’s technique in soleus or longissimus lumbar muscles

**Figure 5:**
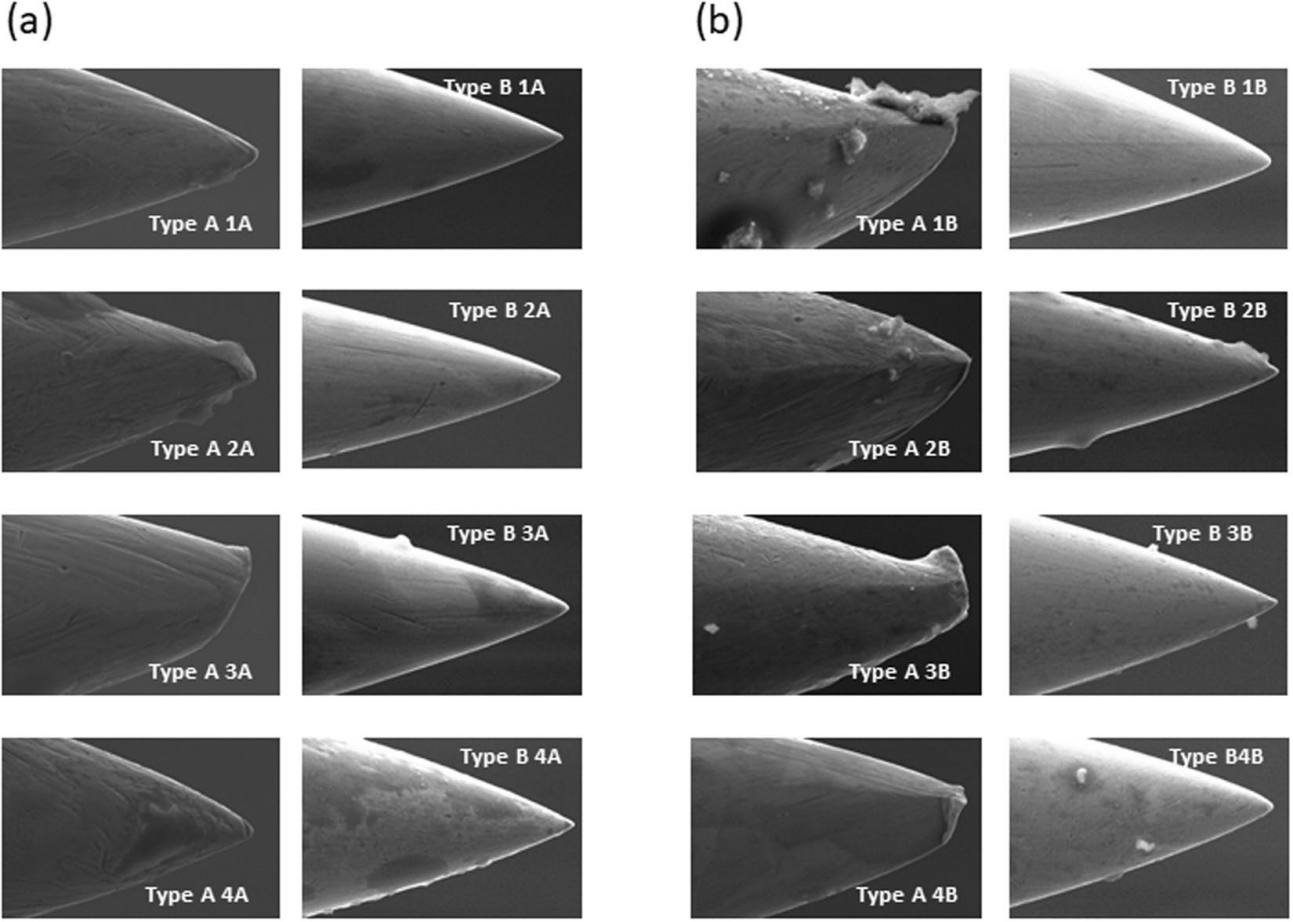
Scanning electronic microscopy images of acupuncture and dry needles after 10 fast in and fast out in soleus and longissimus lumbar muscles. (**a**) Type A1A, Type A2A, Type A3A, Type A4A: Acupuncture needles after 10 fast in and fast out in soleus (magnification 2000x); Type B1A, Type B2A, Type B3A, Type B4A: Dry needles after 10 fast in and fast out in soleus (magnification 2000x). (**b**) Type A1B, Type A2B, Type A3B, Type A4B: Acupuncture needles after 10 fast in and fast out in longissimus lumbar (magnification 2000x); Type B1B, Type B2B, Type B3B, Type B4B: Dry needles after 10 fast in and fast out in longissimus lumbar (magnification 2000x).

The surface conditions of needles after acupuncture manipulation – 10 fast-in and fast-out technique in the soleus or longissimus lumbar– are shown in Fig. 5a,b, respectively. Type B tips were smooth and sharp after performing 10 Hong’s technique in the soleus and longissimus lumbar muscles. In general, the surface characteristics of Type B needles after Hong’s technique were similar to a new Type B needle. Nevertheless, some Type B showed minute scratches, lumps and irregularities (Type B2A, Type B3A, Type B4A, Type B2B, Type B3B and Type B4B) and some needles exhibited cellular tissue adhered to the needle tip surface (Type B4A, Type B2B and Type B3B). This requires further study to see any difference in such interactions from more detailed studies using a SEM beyond imaging, like any tissues attached to the tips and their amounts.

On the other hand, the Type A tip surface showed blunt ends and malformed ends (Type A3A, Type A3B and Type A4B). In general, Type A included more scratches, lumps, irregularities and cellular tissue adhered to the surface (Type A1A, Type A2A, Type A3A, Type A4A, Type A1B, Type A2B and Type A3B) than the Type B.

These results suggest that when we performed deep dry needling in the soleus or longissimus lumbar muscles to evoke the local twitch response^10,20^, the needle tips of both Type B and Type A needles remained in very good condition without significant deformation of the needle tip surface. In clinical practice, Hong’s technique^6^ could be used to evoke local twitch response and the needle tip will remain in similar conditions to those of new needles.

#### After 1–2 inputs or 10 inputs and outputs touching the scapulae bone

**Figure 6:**
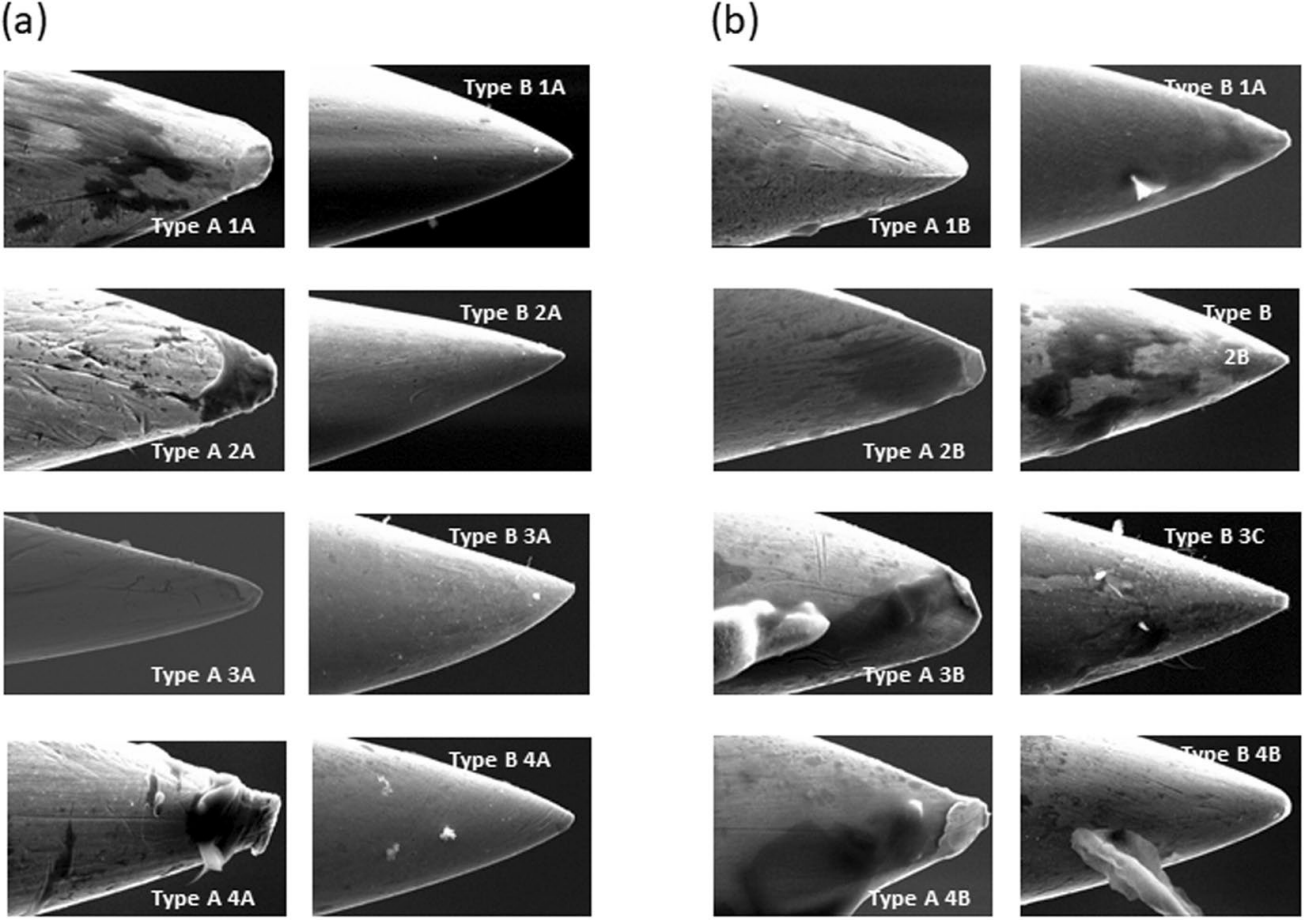
Scanning electronic microscopy images of acupuncture and dry needles after 1–2 and 10 inputs and outputs touching scapulae bone. (**a**) Type A1A, Type A2A, Type A3A, Type A4A: Acupuncture needles after 1–2 inputs and outputs touching scapula bone (magnification 2000x); Type B1A, Type B2A, Type B3A, Type B4A: Dry needles after 1–2 inputs and outputs touching scapula bone (magnification 2000x). (**b**) Type A1B, Type A2B, Type A3B, Type A4B: Acupuncture needles after 10 inputs and outputs touching scapula bone (magnification 2000x); Type B1B, Type B2B, Type B3B, Type B4B: Dry needles after 10 inputs and outputs touching scapula bone (magnification 2000x).

The Type B, after 1–2 inputs touching scapula (Fig. 6a), exhibited a sharp tip with the same characteristics as the new Type B. After 10 inputs in bone (Fig. 6b), the needle tips of Type B needles were very well preserved, although slightly blunt tips were observed. Moreover, Type B exhibited some minute scratches, lumps and irregularities in the needle’s surface (Type B1A, Type B4A, Type B1B, Type B2B, Type B3B and Type B4B). The surface of Type B3B, had cellular tissue adhered.

On the other hand, Type A tips included more important scratches in all needles, showing lumps (Type A2A, Type A4A, Type A1B, Type A3B and Type A4B), irregularities, and cellular tissue adhered to the surface. Finally, we observed blunt and malformed ends (Type A4A, Type A3B and Type A4B).

The inputs performed touching the scapula, indicated to us that Type B should be used in clinical practice when we touch some bone because the needle tip remained better than Type A.

### Qualitative evaluation of both types of needles by professionals

The questionnaire was administered to 40 physical therapists in the state of Spain, 9 women and 31 men; aged 21 to 54 years old with mean age ±SD, 30.6 ± 8.1 years. The mean of number of hours of training in dry needling and/or acupuncture were around 100 hours for each physical therapist, and Hong’s technique^11,17,21^ was the most used technique in clinical practice by physical therapists (n = 32).

The results of the questions asked about the Type B technique (Table 1) indicate that using Hong’s technique it was easier to perform a fast-in and fast-out in clinical practice using Type B, with 57.5% agreeing and 32.5% strongly agreeing than using Type A, with 27.5% agreeing and 2.5% strongly agreeing. Moreover, we asked about ease of penetrating the skin and ease of withdrawing from the skin. The physical therapists answered that Type B needles were easier than Type A needles when penetrating the skin, with 45% strongly agreeing versus 0%. Likewise, when the needles were withdrawn from the skin, the physical therapists answered that Type B were easier than Type A with 17.5% strongly agreeing versus 0%. In clinical practice, Type B seemed easier than Type A when the physical therapist penetrated the skin and when the needle went out the skin. In contrast, Gun’s technique^27^, was performed more easily with Type A with 57.5% strongly agreeing or agreeing versus 42.5% preferring Type B. This result could be because the surface conditions and tip imperfections of Type A facilitate the turning of the needle in catchings muscle fibers and they twist around the needle tip^13^.

**Table 1:** Response percentages for each item of the questionnaire on the Likert scale for Type A and Type B.

Questionnaire	Type of Needle	Strongly agree (%)	Agree (%)	Neither agree nor disagree (%)	Disagree (%)	Strongly disagree (%)
Item 1	Type B	32,5	57,5	2,5	7,5	0
	Type A	2,5	27,5	37,5	30	2,5
Item 2	Type B	15	27,5	30	22,5	5
	Type A	22,5	35	40	2,5	0
Item 3	Type B	22,5	32,5	35	10	0
	Type A	12,5	27,5	55	2,5	2,5
Item 4	Type B	45	22,5	22,5	10	0
	Type A	0	15	30	45	10
Item 5	Type B	17,5	35	40	7,5	0
	Type A	0	20	35	37,5	7,5

Type B: Dry Needling; Type A: Acupuncture Needling. Item 1: Hong’s technique ease to perform a fast-in and fast-out in clinical practice. Item 2: Gunn’s technique: ease to perform a neurological technique. Item 3: Baldry’s technique, ease to perform a superficial technique. Item 4: Ease of penetrating the skin. Item 5: Ease of withdrawing from the skin.

### Limitations of the study

The main drawback of this study is the use only one brand of needles. We have only studied two types of needles from one company (AguPunt) that produces Type B specifically for purpose of dry needling. Other limitation, is that all needles were manipulated by one physiotherapist will produce straight bias technically. Further studies could address this issue using different type of brands and needles in humans.

## Conclusions

In summary, the results of this study show that the tip and the surface conditions of the Type B and Type A from AguPunt brand evaluated with SEM were different in new needles. The tips of Type B were smooth and sharp compared to Type A needles, which had blunt ends. Two types of needles, after performing different needling insertions on human beings, kept the tip of the needle very well preserved, although the Type B suffered very little deformation even when touching the scapula bone 10 times compared to Type A, which deformed slightly. The surface conditions revealed irregularities and scratches in both types of needles but Type A seemed to suffer more scratches and lumps after different needle insertions. The cellular tissue adhered to the surface was similar in both types of needles. The physical therapists’ opinions regarding the use of the two types of needle, Type A and Type B, in clinical practice were that Type B seemed easier than Type A when the physical therapist penetrated the skin and when the needle went out the skin.
